# News and Coming Events

**Published:** 1908-09-26

**Authors:** 


### News and Coming Events.

The old students' dinner of St. Mary's Hospital Medical School will take place at the Whitehall Rooms on Friday, October 2.

Mr. James Reid, of Greenock, left £1,000 to the Greenock Infirmary, £500 to the Eye Infirmary, and £500 to the Greenock Medical Aid and Sick Nursing Society.

The annual dinner of the Past and Present Students of Charing Cross Hospital will take place at the Criterion Restaurant on October 1 under the chairmanship of Mr. Edgar Browne, of Liverpool, the guest of the evening being Sir Patrick Manson.

At a meeting of the University Court of St. Andrews, held on September 19, Mr. Percy J. Herring, assistant in the physiology department of Edinburgh University, was appointed to the new Chandos Chair of Physiology in the United College, St. Andrews.

Mr. E. T. Reed, of *Punch*, will deliver his illustrated lecture "Caricature: In and out of Parliament" to the patients of the Royal Hospital for Incurables, Putney Heath, on the evening of October 12. Mr. Owen Seaman has promised to take the chair.

The International Surgery Congress was opened in Brussels on September 21 in the presence of Prince Albert of Belgium. Professor Cresny, who presided, advocated in his opening speech the creation of an international committee for fighting cancer in all countries.

The annual dinner and first meeting of the Physical Society of Guy's Hospital will be held on October 8. The meeting will be preceded by the dinner, at which Mr. Cosmo Bonsor will occupy the chair. At the meeting Sir R. Douglas Powell, Bart., K.C.V.O., will deliver an address entitled the "Just Procedure of Medicine," under the chairmanship of Dr. Goodhart.

The Third International Congress for the Care of the Insane will be held at Vienna from October 7 to 11, under the patronage of the Austrian Government. The proceedings will deal with all questions relating to the insane in a very comprehensive manner. Among other topics, a report will be read upon the proposal of Dr. Frank, of Zürich, to establish an international institute for the study, prevention, and treatment of mental diseases.

In view of the closing of the old Manchester Royal Infirmary, which will take place shortly, it is proposed to hold a gathering of past and present residents who wish to visit the old building again. A dinner will be held on Thursday, October 15, and the old and new hospitals will be open for inspection on October 15 and 16. Any former resident officer or resident apprentice who has not received notice of the dinner is requested to communicate with the resident medical officer of the infirmary, who is acting as secretary for the gathering.

The King has been graciously pleased to confer the Volunteer Officers' Decoration upon the undermentioned officers of the Volunteer Force, who have been duly recommended for the same under the terms of the Royal Warrant dated July 25, 1892: 2nd Bucks (Eton College) Volunteer Rifle Corps.—Surg.-Major E. S. Norris, M.D. The Queen's Rifle Volunteer Brigade, The Royal Scots (Lothian Regt.).—Surg.-Major J. H. A. Laing, M.B. 1st Dumbartonshire Volunteer Rifle Corps.—Surg.-Lieut.-Col. J. R. F. Cullen, M.D. (retired). 2nd Volunteer Batt. The Welsh Regt.—Surg.-Lieut.-Col. J. A. Jones, M.D.

Mrs. Lister Harland, 117 Bouverie Road, Stoke Newington, N., states that the fund for which she appealed on behalf of Mr. Ernest Harnack, of the X-Ray Department of the London Hospital, has now reached a total of £119 13s. 11d.

Colonel Bruce Vaughan announced another munificent gift to the Cardiff Infirmary at the meeting of the management committee on Sept. 15. He said that a gift of £10,000 had been made to the new wing income fund, and the sum was invested to bring in over £400 per annum. There was no condition attaching to the gift, except that it should be entered as from a Glamorgan coalowner, and that the income derived therefrom should be devoted to the relief of human suffering at the Cardiff Infirmary for all time. This is the third large gift to the Institution in the past few weeks.

The report of the Medical Officer of Health for South Devon for 1907, summarised by Dr. William Harvey (Medical Officer of the Devon County Council), shows that phthisis for the first time caused fewer than 300 deaths; that the deaths from typhoid fever fell to a minimum; and infantile mortality also showed a minimum mortality. The deaths under one year were the fewest on record, while those over sixty-five years were the highest. The birth-rate reached its lowest point in 1907, and the deaths from cancer were more numerous than ever before. For the first time the mortality from cancer was greater than that from phthisis.

On October 1 a meeting of past and present St. George's Hospital students was held in the Medical School, with the object of promoting a St. George's Hospital Club. The club will include the present Amalgamation Club, the Graphic Society, and the Hunterian Society; whilst the *St. George's Hospital Gazette* will become the official organ of the new club, and will in future be forwarded to all members. The objects of the St. George's Club will be to extend and cement the association between all St. George's men, past and present, and to keep them informed of news concerning the hospital and school. Dr. T. T. Whipham, consulting physician to the hospital, has consented to become the first President of the club.

The nomination of the first director of the Australian Institute of Tropical Medicine has been delegated to the Royal Society and to the Liverpool and London Schools of Tropical Medicine. The delegation has been formally accepted by these institutions. Their mode of procedure will be to appoint each a representative, so forming a committee of three, who will carefully consider applications. The actual appointment of the first director, after nomination, will be made by Professor Martin, of the Lister Institute of Preventive Medicine, acting with the Bishop of North Queensland, who initiated and organised this important development in medical science. Professor Martin and Bishop Frodsham act on behalf of the Australian Universities having medical schools, and of the Governments concerned. The Commonwealth Government is subsidising the new Institute with an annual grant of £450, and the Queensland Government with £250 per annum. The Colonial Office has given £400 to the object. Buildings, sufficient for the initiation of the Institute, have been provided in Townsville, North Queensland, which is to be the seat of the Institute.

Mr. Neville Chamberlain presided over a concert at the Central Hall, Birmingham, on September 16, in connection with the Birmingham and Midland Hospital for Women. During an interval in the programme the Chairman moved : "That a Women's Hospital League be formed, with the object, in the first place, of raising £1,000 per annum in aid of the Birmingham and Midland Hospital for Women, and after that has been done, of assisting other hospitals of the city." Mr. John Chamberlain seconded the motion, which was carried with enthusiasm.

Dr. Stephen Wootton Bushell, C.M.G., M.D., B.Sc., M.R.C.S., died recently at Harrow-on-the-Hill. Dr. Bushell qualified in 1866, and in 1868 was appointed physician to H.M. Legation at Pekin, which post he held until 1900. He was much interested in the collection of Chinese curios, books, and coins, and in the study of Chinese art, upon which subject he wrote a handbook. Dr. Bushell was a member of many foreign societies and a Fellow of the Royal Geographical Society. He had formerly been house surgeon at Guy's Hospital and clinical assistant at Bethlem Royal Hospital.

The negotiations which have been in progress for some time between Great Britain and Germany for the conclusion of the agreement to combat sleeping sickness in their African possessions are now practically complete. According to Reuter's Agency, both Governments have agreed to the terms of the convention, although the actual signatures have not yet been affixed. It was originally intended that the new regulations should come into force on October 1, but it is now possible that, in order to allow time for the necessary arrangements to be made locally, they will not become operative until November 1. The convention, which is for a period of three years, provides that British and German doctors and the officials in charge of the concentration camps shall keep in touch with one another to compare the result of their various researches. Segregation camps will be established on either side of the international boundary, while infected natives will be prevented from passing into uninfected districts, such persons being detained and segregated. The convention also provides for the notification to the officials of both Governments of all infected areas, and for taking effective measures for dealing with crocodiles or other animals which may be found to be the food of the fly which carries the disease.

The report submitted at the quarterly Court of the Governors of the London Hospital held on September 2, showed that, in response to the quinquennial appeal, a sum of £64,798 had been received, and contributions amounting to £8,968 had been promised. By a collection made on the Stock Exchange £5,700 had been realised. In connection with the Samuel Lewis bequest, application had been made for £25,000 Great Western and Great Central Railways guaranteed stock, but only £6,000 of these stocks had been allotted to the hospital. The committee recorded with regret the deaths of Mr. G. C. Croft, who had shown a keen interest in the work of the hospital, and Mr. H. L. Barnard, in whom the hospital staff had lost an indefatigable worker and an able surgeon. Several alterations and improvements had been made in the internal administration of the hospital. In moving the adoption of the report, the chairman, Mr. C. H. Hale, said thanks were due to the Lord Mayor, who gave a dinner on behalf of the funds of the hospital, by which a substantial sum was obtained, and to Mme. Melba, for organising a concert, as a result of which £2,000 was received towards the funds of the hospital. The amount realised so far as the outcome of the quinquennial appeal was very satisfactory.

Dr. S. Weir Mitchell, of Philadelphia, has been elected a foreign Fellow of the Royal Society of England. Benjamin Franklin, we learn from the New York "Medical Record," was the first American to be elected a member. Only three Americans are now on its roll—Alexander Agassiz, George W. Hill, and Simon Newcomb.

A committee appointed by the Medico-Surgical Society of Brabant for the study of cancer in Belgium met at Brussels last week under the presidency of Dr. Vince. In his inaugural address, the Chairman announced that at his suggestion the Council of the Brussels Hospital had decided to create a special branch for the treatment of cancer, while the committee itself would undertake the work of collecting statistics relating to the number of cases of, and percentage of deaths from, cancer throughout the country and such other data as would enable them to draw up tables giving the geographical distribution of cancer, its principal centres of activity, and details regarding contagion, transmission, and heredity.

